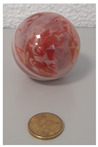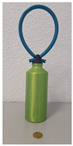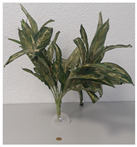# Control Strategy of an Underactuated Underwater Drone-Shape Robot for Grasping Tasks

**DOI:** 10.3390/s22228828

**Published:** 2022-11-15

**Authors:** Juan S. Cely, Miguel Ángel Pérez Bayas, Marco Carpio, Cecilia Elisabet García Cena, Avishai Sintov, Roque Saltaren

**Affiliations:** 1 Centro de Automática y Robótica, Universidad Politécnica de Madrid, C/José Gutiérrez Abascal 2, 28006 Madrid, Spain; 2 Escuela Superior Politécnica de Chimborazo, Riobamba 060155, Ecuador; 3 Grupo de Investigación en Interacción Robótica y Automatica (GIIRA), Universidad Politécnica Salesiana, Calle Turuhayco 3-69 y Calle Vieja, Cuenca 010105, Ecuador; 4 Escuela Técnica Superior de Ingeniería y Diseño Industrial, Universidad Politécnica de Madrid, Ronda de Valencia 3, 28012 Madrid, Spain; 5 School of Mechanical Engineering, Tel-Aviv University, Tel-Aviv Yafo 6226414, Israel

**Keywords:** agricultural automation, field robots, grasping, marine robotics, mobile manipulation, underactuated robots

## Abstract

In underwater environments, ensuring people’s safety is complicated, with potentially life-threatening outcomes, especially when divers have to work in deeper conditions. To improve the available solutions for working with robots in this kind of environment, we propose the validation of a control strategy for robots when taking objects from the seabed. The control strategy proposed is based on acceleration feedback in the model of the system. Using this model, the reference values for position, velocity and acceleration are estimated, and then the position error signal can be computed. When the desired position is obtained, it is possible to then obtain the position error. The validation was carried out using three different objects: a ball, a bottle, and a plant. The experiment consisted of using this control strategy to take those objects, which the robot carried for a moment to validate the stabilisation control and reference following the control in terms of angle and depth. The robot was operated by a pilot from outside of the pool and was guided using a camera and sonar in a teleoperated way. As an advantage of this control strategy, the model upon which the robot is based is decoupled, allowing control of the robot for each uncoupled plane, this being the main finding of these tests. This demonstrates that the robot can be controlled by a control strategy based on a decoupled model, taking into account the hydrodynamic parameters of the robot.

## 1. Introduction

Underwater tasks are commonly related to pulling out objects from the seabed, waste cleaning, rescuing objects and/or people, underwater manufacturing, and construction and mining [1,2]. These tasks usually require a mobile platform to approach and interact with the underwater environment or with the objects. However, such platforms face various challenges, including securing their position in restless water and remote operation in the deep ocean [3,4]. For some time now, the scientific community has been proposing various systems, referred to as underwater vehicle manipulator systems (UVMS) [5]. A UVMS is a mobile platform with manipulation capabilities aimed at meeting the needs of the maritime industry [6].

In [7,8], solutions for problems such as exploring the seabed, collecting objects, and assisting divers in industrial underwater scenarios are given. For these activities, the mobile platform should have the technical characteristics required to hold their position and to manipulate different objects in underwater conditions. These conditions could be different from those required by the grounded robots, e.g., in terms of sea currents, leading to limitations in the use of vision and perception algorithms, as shown in [9,10,11]. The demand for a control strategy to guarantee the stability and the position of the robot represents a challenge to the technical proposal during the design process for this kind of robot.

The controller must be able to guarantee the position of the robot, which is important for the manipulation process. The interaction with the environment is a relevant challenge that has been previously described, as shown in [12]. Taking into account the problem of grasping in underwater environments, our research group designed and patented a robot with two arms as an approach to this issue [13]. The proposed robot has a special complexity in terms of the mechanical structure, having a modular design and two arms with more than three degrees of freedom (DOF) per arm.

**Figure 1:**
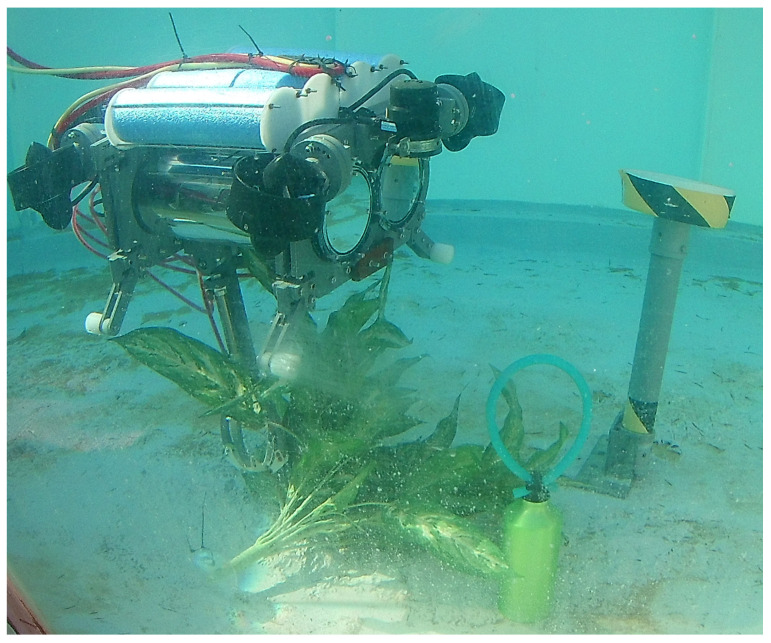
The UDrobot picking up a plant in an underwater environment.

Based on this proposal, we decided to redesign the underwater modular drone, simplifying its design and adapting the fabrication to be as cheap as possible. In the process, we created *UDrobot*, short for underwater drone robot, which can be seen in Figure 1. UDrobot is an underactuated underwater robot which has four thrusters, similar to an aerial drone, tethered with two lines: one for data and another one for powering. The combined force from the four oriented thrusters enables its spatial motion in SE(3); however, the number of actuators is less than the robot’s DOF, and so the robot has the condition of being an underactuated system. Furthermore, the UDrobot has one DOF arm mounted on its bottom which allows interaction with the underwater environment, as is recommended in the previous literature.

As mentioned above, the robot has four thrusters allowing it to be driven. A sufficiently detailed model development was carried out, similar to the one presented by Alkamachi [14], where a controller for an air drone with thrusters is proposed with a light inclination to improve the contribution of the thrust around the vertical angle. For this strategy, it is required to know the model of the robot, which can be decoupled into two subsystems: the altitude system and the attitude system. However, for underwater systems, this could be a problem because the coupled effects are more significant according to the physical parameters or the current state of the system. To obtain a model that is more faithful to reality, an identification process developed by the research team at Cely was carried out [15]. Another method that can be used for the identification of hydrodynamic parameters was developed by Gibson [16], validated with real robots. When the hydrodynamic parameters are known, the systems could be modelled in more detail, which would allow new model-based control strategies to be proposed.

The robot having the condition of being underactuated raises a specific scheme in the control condition, which drones also have in the air. This process was also developed by Chocron, where, according to the structural variation in the thruster allocation, the matrix achieves a better approximation for each desired case [17]. Another approach was proposed by Doniec [18], suggesting the reconfiguration of the thrusters to achieve a greater incidence of forces in each of the components of a SE(3) group. A process similar to that proposed by us was developed by Jin [19,20], where a quadrotor with inclined thrusters was controlled. These references show how the process for air drones has been carried out by different research teams, but the process for underwater systems has not yet been addressed in the same way, this being the field in which our research group has worked for more than fifteen years.

As already described, UVMS have become a popular case study, especially when it is necessary to conduct the analysis and control of robotic manipulators, for the specific case when the robot used is in the form of a drone. Simetti provided a description of the UVMS and discussed the challenges to be overcome, such as the control and planning of the trajectories [6]. Another development was described by Jiao [21], where a drone is controlled with a robotic arm suspended from it, and its robustness is analysed. Knowing the model and the conditions that make the UDrobot a UVMS, we propose simplifying the physical model to be controlled with strategies that are different to those proposed in the literature, based on physical characteristics, achieving subsystems that allow the model to be analysed and controlled in a more direct way. A specific case was developed by Barbalata [22], where the decoupling of a physical model for a UVMS was proposed. With a network of sensors on the surface of the robot, Nelson managed to measure the hydrodynamic forces on the rigid body and proposed a decoupling according to the measured values [23]. When the dynamics of a system model are highly coupled, that is to say, the effects on one analysis plane are indirectly perceived in another, a decoupling method can be developed, which Nielsen described in the research [24].

In the case of control in this type of system, it is possible to achieve control using conventional strategies, but one controller is required for each generated subsystem. Garcia proposed a system based on sliding mode control that manages to eliminate the disturbances, but to be able to adapt to these disturbances, a neural network must be used to re-tune the controller’s gains [25]. On the other hand, Labbadi managed to achieve decoupled control in a quadrotor, but it only decouples in two subsystems: altitude and attitude [26].

To solve the various problems experienced by the controllers, the scientific community has proposed improvements to each. To eliminate the effects of the load on the control, Perez proposed an adaptive control to be able to compensate for this value [27]. Manzanilla was more focused on the pilot as a source of error when teleoperating the system, and proposed a certain degree of autonomy for manipulation tasks using artificial vision [28]. Without a doubt, the system can track trajectories if it has a controller that guarantees the desired position, and this can be achieved with a configuration such as the one that has been proposed; this was validated by Yahya’s research [29]. The systems to operate the manipulators are usually composed of electrical motors, as shown by Skaldebo [30]; however, for this case, the method used to transfer the power is based on our patent to transfer movement [31].

This article seeks to demonstrate the implementation of a control scheme that uses the acceleration feedback, applied to each one of the decoupled subsystems to reduce the complexity of the model. The results obtained allow the recognition of this type of control as valid for underwater grasping tasks. The detailed development of each of the components of the robot dynamic model had to be carried out, and, according to various physical considerations, a set of four models decoupled from each other was proposed. After this, the implementation of a controller based on previous developments was achieved, and the values of the hydrodynamic coefficients obtained from experiments prior to this control were integrated. For the validation of the system, a methodology was carried out in which an object was captured, lifted, and transported on a route for which the pilot had one decision to make. The items that were chosen to be captured and carried by the robot were a ball, a metal bottle, and a plant. For each of the objects, a description of the results is provided, allowing the comparison of the effects generated by the load of each of the elements to be carried, including the effects of the acceleration on the angles and the control action around the yaw angle.

The article is structured as follows: We begin with the detailed development of the model, where clarity is provided on each of the components that can affect the dynamics. After this, a description of the proposed simplified model is given. Then, a description of the controller is given based on the acceleration feedback, denoting how to obtain its gains to ensure convergence. The following is a description of the methodology implemented to carry out each experiment, with each of the stages proposed for this purpose. The following section describes the results obtained from the experimentation. The final section is dedicated to discussing the results obtained, and some conclusions are drawn according to the objectives.

## 2. Modelling of the Underwater Robot

**Figure 2:**
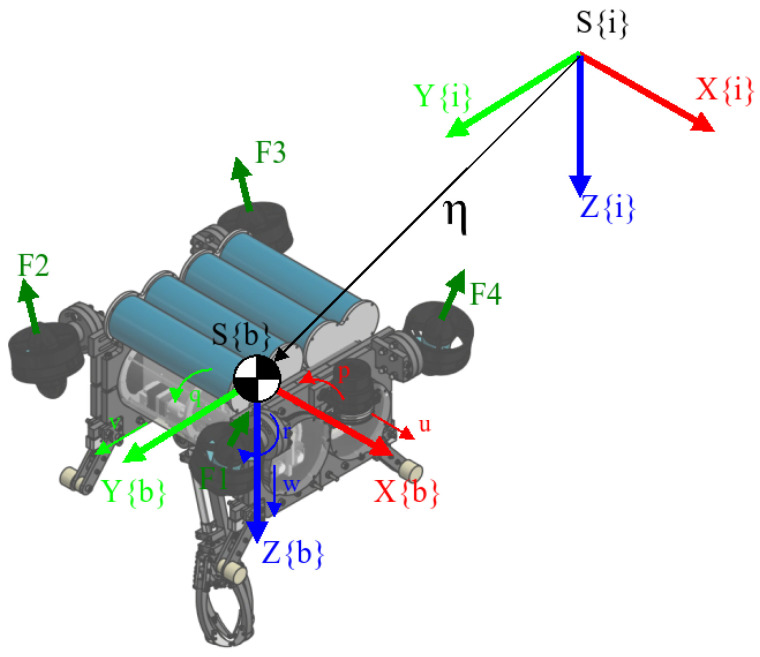
Relationship between the inertial-fixed frame S{i} and body-fixed frame S{b}. The forces on the thruster are the generated push for each one.

UDrobot is an underwater drone-shaped robot designed to carry out various tasks. It has an arm with one DOF on its underside that can be used for pulling out objects from the seabed. The robot is composed of two waterproof enclosures containing electronics relative to the control of the robot and the electronic control board for the arm. These enclosures are held in two frames that support the arm and the thrusters. The robot is drone-shaped in that it has four T200 thrusters from BlueRobotics, oriented upward with a low frontward orientation for the thruster on the front side and a backward orientation for thrusters on the backside. This generates an effect in more than one axis, as described in Section 2.5. For a complete description of the elements used in the modelling, Figure 2 shows the nomenclature for each axis. The angles are measured with an inertial unit measure (IMU) on an autopilot board in the robot, where there is a depth sensor. Communications and power are carried through the robot by a pair of cables.

The inertial-fixed frame is given by S{i}, where the position and orientation of the UDrobot are denoted by $\eta_{1}={\left(x,y,z\right)}^{T}\in R^{3}$ and $\eta_{2}={\left(\phi ,\theta ,\psi \right)}^{T}$, respectively. We denote the position relative to S{i} by the general form $\eta =[\eta_{1}^{\prime },\eta_{2}^{\prime }]$. While the velocity is described on S{b}∈SE(3) using a SNAME (Society of Naval Architects and Marine Engineers) convention, where $\nu_{1}={\left[u,v,w\right]}^{\prime }$ with translational velocities in x,y and *z* for the body-fixed frame correspondingly, for the angular velocities, the description is based on $\nu_{2}={\left[p,q,r\right]}^{\prime }$ being the angular velocity for each axis.

### 2.1. 6-Dof Kinematics

There are different options for referring to the orientation of the rigid body in an inertial-fixed frame. For this case, we use a rotation matrix composed of Euler angles ($\eta_{2}$). For this representation, we use $c_{\alpha }$ as cos(α) and $s_{\alpha }$ as sin(α), with each α being a Euler angle. The rotation matrix $R_{I}^{B}\left(\eta_{2}\right)$ from the inertial frame to the body frame is described in (1).

(1) $$R_{I}^{B}\left(\eta_{2}\right)=\left[\begin{array}{ccc} c_{\psi }c_{\theta } & s_{\psi }c_{\theta } & -s_{\theta }\\ -s_{\psi }c_{\phi }+c_{\psi }s_{\theta }s_{\phi } & c_{\psi }c_{\phi }+s_{\psi }s_{\theta }s_{\phi } & s_{\phi }c_{\theta }\\ s_{\psi }s_{\phi }+c_{\psi }s_{\theta }c_{\phi } & -c_{\psi }s_{\phi }+s_{\psi }s_{\theta }c_{\phi } & c_{\phi }c_{\theta } \end{array}\right]$$

The rotational matrix is used to transform the velocities into the derivates of the position; nevertheless, to convert angular velocities to angular derivates, a matrix is required, described by $T\left(\eta_{2}\right)\in R^{3\times 3}$, where the structure is shown in (2).

(2) $$T\left(\eta_{2}\right)=\left[\begin{array}{ccc} 1 & 0 & -\sin(\theta )\\ 0 & \cos(\phi ) & \cos(\theta )\sin(\phi )\\ 0 & -\sin(\phi ) & \cos(\theta )\cos(\phi ) \end{array}\right]$$

To obtain a bigger matrix that relates the transformation between the referred body velocities and the position derivates, let us define $J\left(R_{I}^{B}\right)\in R^{6\times 6}$ as a matrix for which the structure is composed of the rotational matrix and an angular velocity transformation matrix ($T(\eta_{2})$), as defined in (3):

(3) $$J\left(R_{I}^{B}\right)=\left[\begin{array}{cc} R_{I}^{B}\left(\eta_{2}\right) & O_{3x3}\\ O_{3x3} & T(\eta_{2}) \end{array}\right]$$

Finally, the transformation could be defined as shown in (4):

(4) $$\nu =J\left(R_{I}^{B}\right)\dot{\eta }$$

### 2.2. Dynamics Modelling

The dynamic model of a rigid body could be defined by how the behaviour of the body is reflected in the relationship between forces and torques, with kinematic elements being fundamental parts of this description. In this case, the analysis was carried out by taking into account the hydrodynamic effects, which we will describe. The general nature of the dynamic model described by [32] can be seen in (5), where *F* is the forces vector and τ is the torques vector, $r_{com}$ is the distance between the centre of mass and the body-fixed frame, and $I_{o}$ is the inertial tensor:

(5) $$\begin{array}{c} F=m\{\dot{\nu_{1}}+\left(\nu_{2}\times \nu_{1}\right)+\left(\dot{\nu_{2}}\times r_{com}\right)+\nu_{2}\times \left(\nu_{2}\times \right)r_{com}\}\\ \tau =I_{o}\dot{\nu_{2}}+\left(\nu_{2}\times \left(I_{o}\nu_{2}\right)\right)+mr_{com}\times \left(\dot{\nu_{1}}+\nu_{2}\times \nu_{1}\right) \end{array}$$

However, the dynamic model could be rewritten in different ways. We use a parametrisation for the rigid body as an equation of motion with mass effects and Coriolis effects, both of them equal to forces and torques. The first element in the dynamic model is the effect generated by the mass and inertia. Let us define the *mass matrix* or *inertial matrix* as shown in (6), where *S* means a *skew-antisymmetric matrix*:

(6) $$M_{RB}=\left[\begin{array}{cc} mI_{3} & -mS(r_{com})\\ mS(r_{com}) & I_{o} \end{array}\right]$$

where *m* is the body mass, $I_{3}$ is an identity matrix of the third order and $I_{o}$ is the inertial tensor. For the Coriolis effect, we use a parametrisation based on the mass matrix as defined in (7), where the submatrices $M_{ij}\in R^{3\times 3}$ are based on the mass matrix:

(7) $$C_{RB}=\left[\begin{array}{cc} 0_{3\times 3} & S(M_{11}\nu_{1}+M_{12}\nu_{2})\\ -S(M_{11}\nu_{1}+M_{12}\nu_{2}) & S(M_{21}\nu_{1}+M_{22}\nu_{2}) \end{array}\right]$$

One way to describe the dynamic behaviour of the rigid body is to use the equation of motion (8), which is a relevant way to describe the relationship between the forces and physical parameters of the rigid body. In this case, we define the gravitational vector (g(η)) as a product of the hydrostatic force, which will be described in another subsection. On the other hand, the hydrodynamic forces vector is taken as a load force vector, which will also be defined in the next subsection:

(8) $$M_{RB}\dot{\nu }+C_{RB}\left(\nu \right)\nu +g\left(\eta \right)=\tau +\tau_{hdy}$$

### 2.3. Hydrodynamics Effects

The hydrodynamic effects are the effects generated by a fluid when a submerged rigid body is moving within it. The most important hydrodynamic effects are added mass and viscous damping. The added mass can be defined as a virtual mass that generates more mass on the rigid body, with these values denoted by $M_{A}\in R^{6\times 6}$, defined for translational displacement using a coefficient ($C_{mi}$), where *i* means the axis of the coefficient, and for angular displacement as the double of the inertial value for this angle:

(9) $$M_{A}=diag\left\{C_{mx}\rho \nabla ,C_{my}\rho \nabla ,C_{mz}\rho \nabla ,I_{XX},I_{YY},I_{ZZ}\right\}$$

where ρ is the fluid density, ∇ is the volume of the rigid body, and $I_{ii}$ represents each diagonal component of the inertial tensor. For the Coriollis case, we can denote this as $C_{A}\in R^{6\times 6}$, and its structure is based on the parametrisation in the rigid body, as shown in (10):

(10) $$C_{A}=\left[\begin{array}{cc} 0_{3\times 3} & -S(A_{11}\nu_{1}+A_{12}\nu_{2})\\ -S(A_{11}\nu_{1}+A_{12}\nu_{2}) & -S(A_{21}\nu_{1}+A_{22}\nu_{2}) \end{array}\right]$$

where the submatrices $A_{ij}\in R^{3\times 3}$ are based on the added mass matrix. The representation of these effects is achieved in the same way as in rigid body modelling. The second effect is viscous damping, the best known representation of which is the drag force. In this case, we select a coefficient to be packaged into the matrix representation, as shown in (11):

(11) $$\begin{array}{c} D\left(\nu \right)=diag\{K_{qu}|u|,K_{qv}|v|,K_{qw}|w|,K_{qp}|p|,K_{qq}|q|,K_{qr}|r|\}\\ F_{drag}=D\left(\nu \right)\nu \end{array}$$

where $K_{qi}$ is the quadratic coefficient for each axis, which is multiplied by the absolute velocity on the axis. The values for added mass and for viscous damping are obtained as described in [15].

### 2.4. Gravitational and Buoyancy Effects

The gravitational effect is present when the rigid body is under the effects of a gravitational field; however, in this case there is another effect, which is a function of the gravity acceleration: the buoyancy. For modelling these effects, we define weight as $W=me_{3}\hat{g}$ and buoyancy as $B=\rho \nabla e_{3}\hat{g}$, where *m* is mass, $\hat{g}={\left[0,0,-9.81\right]}^{T}$ is gravity acceleration in vector form, ρ is fluid density, ∇ is the volume of the rigid body, and $e_{3}$ is a unit vector facing downward. The force and torque vector generated are shown in (12) and (13), respectively:

(12) $$f_{G}\left(R_{I}^{B}\right)=R_{I}^{B}\left[\begin{array}{c} 0\\ 0\\ W \end{array}\right]$$

(13) $$f_{B}\left(R_{I}^{B}\right)=R_{I}^{B}\left[\begin{array}{c} 0\\ 0\\ -B \end{array}\right]$$

To merge both effects into a simple vector in order to then integrate it into the equation of motion, we can rewrite the gravitational effect. In this case, we define $r_{com}$ as the distance from the centre of mass to the body-fixed frame and $r_{cob}$ as the distance from the centre of buoyancy to the body-fixed frame. The vector description is shown in (14):

(14) $$g\left(R_{I}^{B}\right)=-\left[\begin{array}{c} f_{G}\left(R_{I}^{B}\right)+f_{B}\left(R_{I}^{B}\right)\\ r_{com}\times f_{G}\left(R_{I}^{B}\right)+r_{cob}\times f_{B}\left(R_{I}^{B}\right) \end{array}\right]$$

### 2.5. Thruster Allocation Matrix

**Figure 3:**
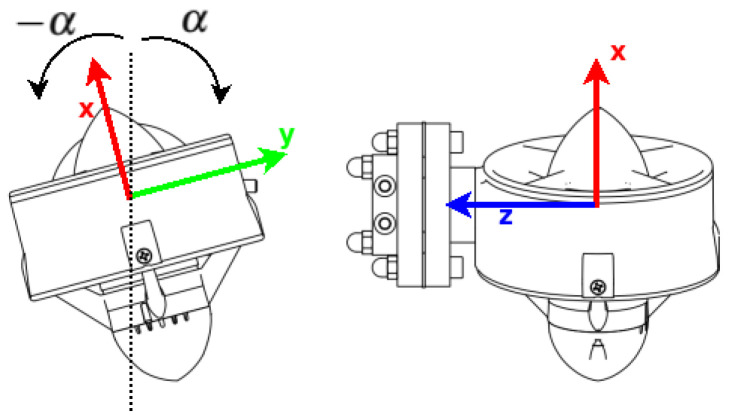
Angle of inclination per each thruster. The value of α is constant for the experiment at 5 degrees.

The allocation matrix is the name of the matrix that relates the input values with the effects per each degree of freedom. In the case of the drones, this matrix is also called the *mixer matrix* or *thruster allocation matrix*. For this specific case, we have to remember the orientation of each thruster. In Figure 3, we can see the angle and how it modifies the thrust orientation.

The allocation matrix is denoted by $B\in R^{6\times 4}$, where its structure is visible in (15). In this case, this matrix has two special characteristics: the dimensions of the matrix are the number of degrees of freedom and the number of inputs to the system, this being the most important characteristic because it defines the system as an *underactuated system*; the other characteristic is that it is a function of α. However, the values are constants and they do not change if the orientation of the thruster does not change; here, for every experiment, the value was constant at 5 degrees. The values of $\bar{x}$ and $\bar{y}$ are the perpendicular distance between the thruster location and the denoted axis:

(15) $$B\left(\alpha \right)=\left[\begin{array}{cccc} -\sin(\alpha ) & \sin(\alpha ) & \sin(\alpha ) & -\sin(\alpha )\\ 0 & 0 & 0 & 0\\ \cos(\alpha ) & \cos(\alpha ) & \cos(\alpha ) & \cos(\alpha )\\ -\cos\left(\alpha \right)\bar{y} & -\cos\left(\alpha \right)\bar{y} & \cos\left(\alpha \right)\bar{y} & \cos\left(\alpha \right)\bar{y}\\ \cos\left(\alpha \right)\bar{x} & -\cos\left(\alpha \right)\bar{x} & -\cos\left(\alpha \right)\bar{x} & \cos\left(\alpha \right)\bar{x}\\ \sin\left(\alpha \right)\bar{y} & -\sin\left(\alpha \right)\bar{y} & \sin\left(\alpha \right)\bar{y} & -\sin\left(\alpha \right)\bar{y} \end{array}\right]$$

### 2.6. Matrix Form

A compact method to represent the dynamic behaviour of the rigid body is to join every term analysed in this section, such as mass and Coriolis in a rigid body, hydrodynamic effects and gravitational effects, and for the required forces, the allocation matrix and the system input. Let us define $M=M_{RB}+M_{A},\;M\in R^{6\times 6}$ and $C=C_{RB}+C_{A},\;C\in R^{6\times 6}$; we can then define a matrix form, as is shown in (16), where *u* is the input vector, that for this case is $u\in R^{4\times 1}$:

(16) $$M\dot{\nu }+C\left(\nu \right)\nu +D\left(\nu \right)\nu +g\left(R_{I}^{B}\right)=Bu$$

However, for determinate cases, we need the model to refer to the inertial-fixed frame. To conduct this transformation, we have to multiply each element referring to the body-fixed frame by a rotation matrix, which adds complexity to the model, making its analysis more difficult. The components relative to the mass matrix and Coriollis matrix have a large number of cross terms, meaning that there are many terms which can have effects in an axis different to the one being analysed. If certain physical conditions are satisfied, it is possible to disengage the model, simplifying its analysis.

## 3. Simplified Model

According to Fossen [33], if the robot has a symmetric shape and the distribution of mass is uniform, it is possible to disengage the model in different subsystems. For this case, it was disengaged in three subsystems. The first is the lateral subsystem, where the components related to the X axis and Z axis are analysed, while the second obtained subsystem is the frontal subsystem, where it is possible to see the components above the Y axis and Z axis. The last subsystem that we can define is the normal subsystem, where it is possible to see the X–Y plane. All of the forces and torques obtained from those schemas are based on the body-fixed frame, and it is possible to work with this model if the robot has a low velocity and we can assume $\dot{\eta }\approx \nu$ and the value for $r_{cob}={\left[0,0,0\right]}^{T}$, meaning that it coincides with the body-fixed frame. For localisation tasks, values must be transformed to the inertial-fixed frame. Although there are thruster forces in the diagram, these are not generated cross effects; thus, we can keep using the matrix *B* described in Section 2.5.

### 3.1. Lateral Subsystem

**Figure 4:**
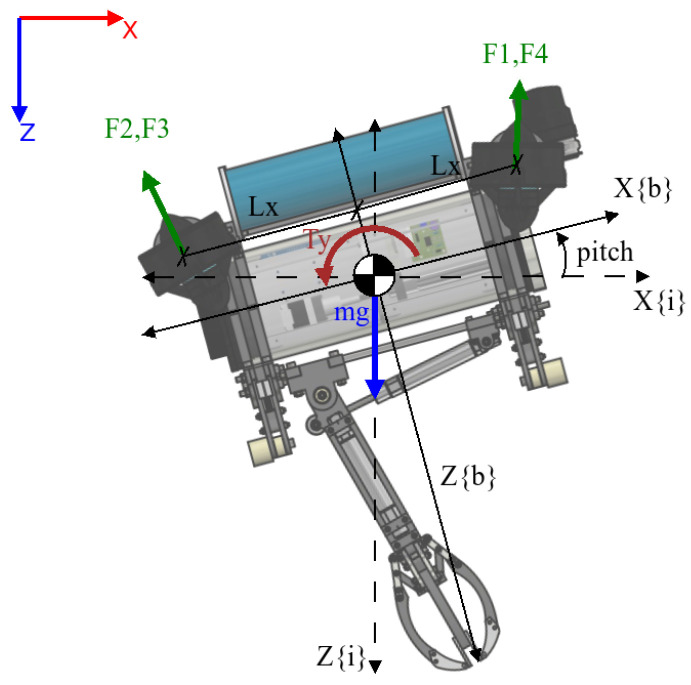
The applied forces visible on the X–Z plane of the body-fixed frame.

The lateral subsystem is based on the X–Z plane, as shown in Figure 4, where it is possible to model the angular displacement in the pitch angle (θ) with an input $\tau_{Y}$. We can make this assumption under the condition that the value for the pitch angle will be close to zero.

The formulation for this subsystem is visible in (17), $r_{com_{X}}$ being the component of the buoyancy in the X axis:

(17) $$\tau_{Y}=2I_{YY}\ddot{\theta }-K_{qq}\left|\dot{\theta }\right|\dot{\theta }-r_{com_{X}}mg$$

**Figure 5:**
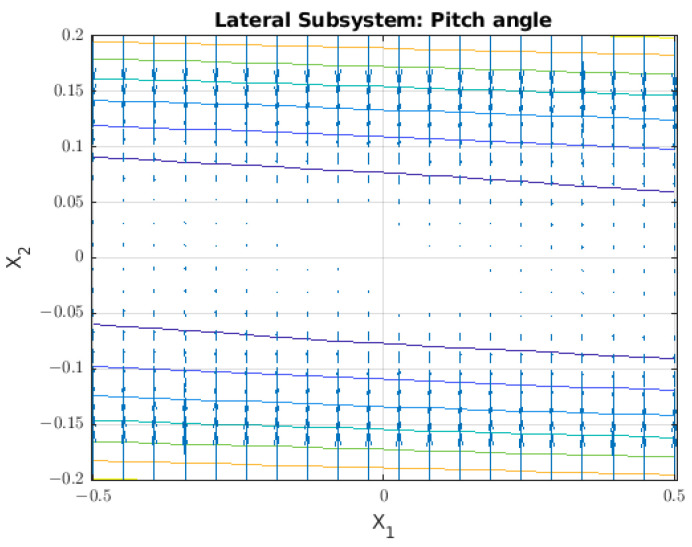
Vector field for the lateral subsystem where the phase variables are $\theta =x_{1}$ and $\dot{\theta }=x_{2}$.

We graph the behaviour of the subsystem in a vector field, as shown in Figure 5, and we find that by this graphical method, the system has an equilibrium point when the $\dot{\theta }=0$ for any value of θ.

For this subsystem, it is possible to obtain the relationship between *Z* and the value for the force ($F_{Z}$), which is visible in (18):

(18) $$F_{Z}=\left(m+C_{mz}\rho \nabla \right)\ddot{Z}-K_{qz}\left|\dot{Z}\right|\dot{Z}-\left(mg-B\right)$$

**Figure 6:**
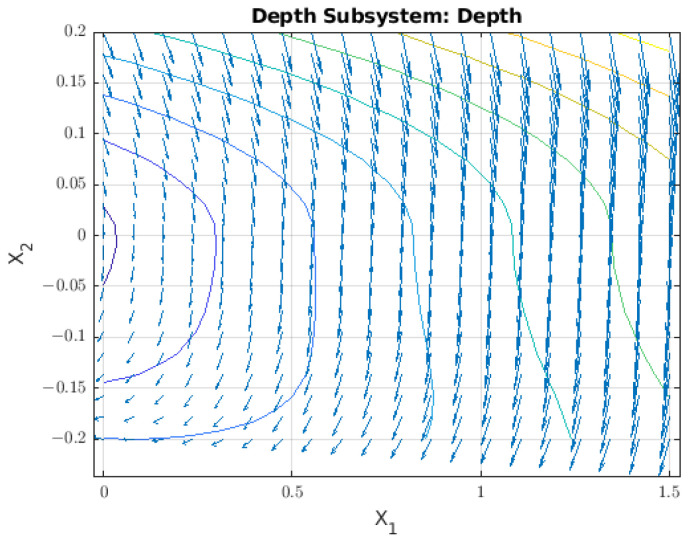
Vector field for displacement in the *Z* axis where the phase variables are *Z* and $\dot{Z}$. The limits for *Z* begin at zero, as this measurement represents depth.

With respect to the dynamic behaviour, the *Z* axis is graphed as a vector field and is shown in Figure 6.

The vector field for the dynamic behaviour of the robot in the *Z* axis shows a slight displacement independent of the point where the robot was located, and there is also an equilibrium point at zero. With this information, we can define the robot as having *neutral buoyancy*. A physical way to determinate this is if the rest of weight *W* and the buoyancy *B* are close at zero.

### 3.2. Frontal Subsystem

**Figure 7:**
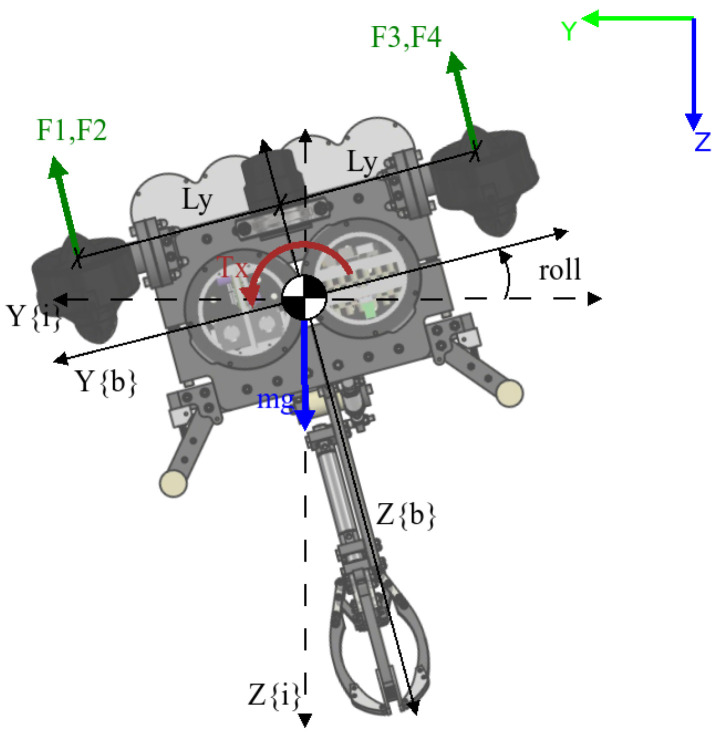
The applied forces visible on the Y–Z plane of the body-fixed frame.

The frontal subsystem shows the Y–Z plane of the robot. From this schema, it is possible to obtain the values for $\tau_{X}$ and the angular displacement of the roll angle (ϕ). In Figure 7, it is shown the diagram of forces applied to the robot from this perspective.

The obtaining of $\tau_{X}$ is shown in (19), where $r_{com_{Y}}$ is the component on the Y axis of the centre of buoyancy.

(19) $$\tau_{X}=2I_{XX}\ddot{\phi }-K_{qp}\left|\dot{\phi }\right|\dot{\phi }-r_{com_{Y}}mg$$

**Figure 8:**
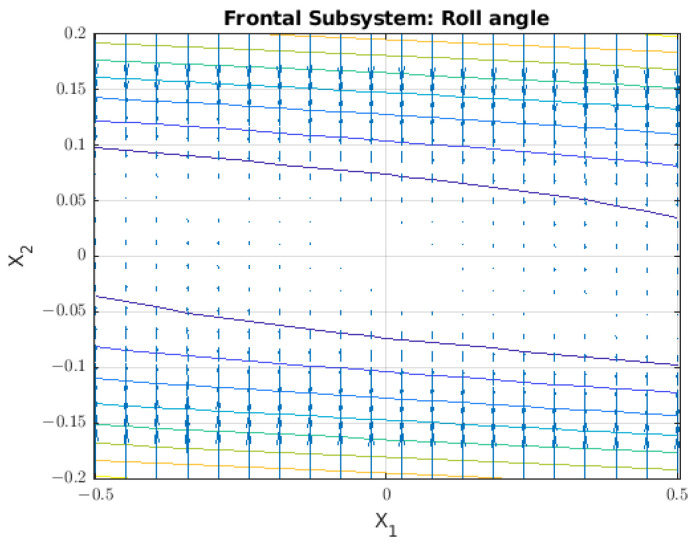
Vector field for the lateral subsystem where the phase variables are $\phi =x_{1}$ and $\dot{\phi }=x_{2}$.

For analysing the behaviour of the frontal subsystem, the vector field for this subsystem is shown in Figure 8.

In this case, when the velocity is zero, the vector field shows that the subsystem remains near at the equilibrium point for any value of the angle. This characteristic is relevant for linearising systems due to equilibrium points around where a line of stable points is generated. Despite that, our system is non linear, and its dynamics depends on initial conditions and other parameters, such as hydrodynamic coefficients and localization of points as centre of mass and centre of buoyancy; therefore, the line of stable points around velocity zero is not a sufficient condition to guarantee the stability of the entire system.

### 3.3. Normal Subsystem

**Figure 9:**
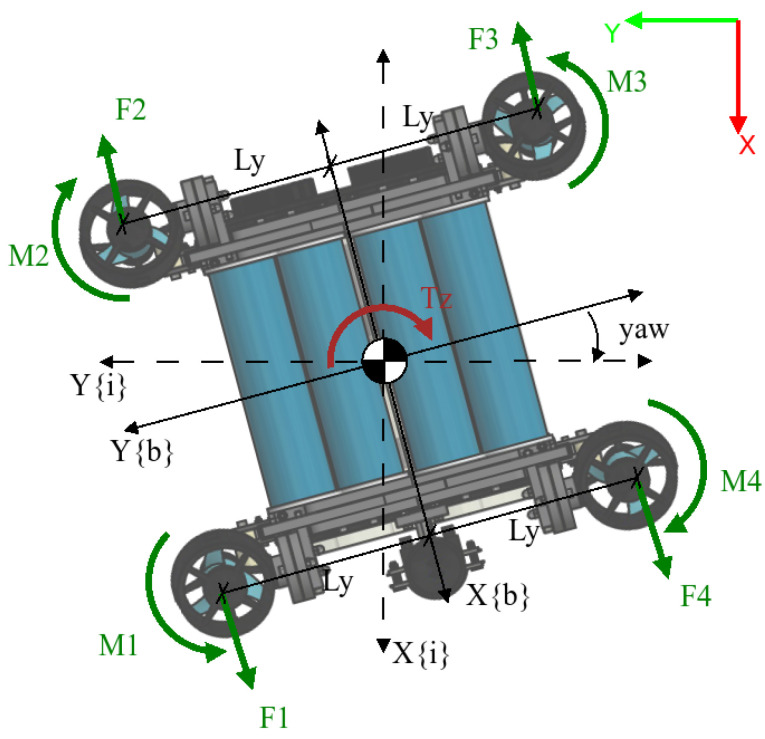
The applied forces visible on the X–Y plane of the body-fixed frame.

The normal subsystem shows the component on the X–Y plane (Figure 9). In this case, it is possible to model the torque ($\tau_{Z}$) and the yaw angle (ψ). In the corresponding diagram, it is possible to see the moments generated by the thruster, but their values are low and they are neglected.

The formulation for this case is shown in (20), where the value for the $\tau_{cable}$ is unknown and is a function of the position of the cables:

(20) $$\tau_{Z}=2I_{ZZ}\ddot{\psi }-K_{qr}\left|\dot{\psi }\right|\dot{\psi }-\tau_{cable}$$

**Figure 10:**
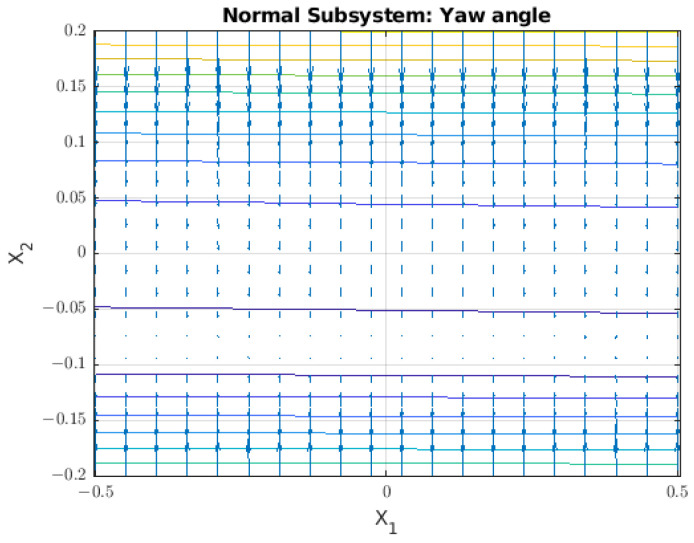
Vector field for the lateral subsystem where the phase variables are $\psi =x_{1}$ and $\dot{\psi }=x_{2}$. The value for $\tau_{cable}$ was selected to be 1 Nm.

Continuing with the analysis of the system using a vector field, this vector field is visible in Figure 10.

In the case of the normal subsystem, we find that it also has an equilibrium point where $\dot{\psi }$ is zero, no matter the value of angle (ψ).

## 4. Control Strategy

**Figure 11:**
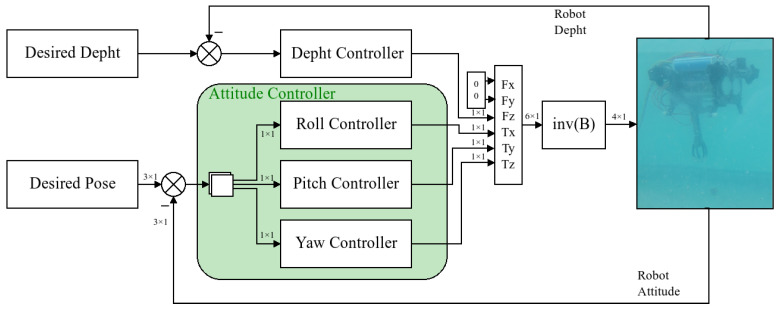
Control schema describing the disengagement of controllers, where *B* is the thruster allocation matrix, and the desired position is in Euler angles.

Due to the equilibrium points for each subsystem, we propose that the system could be controlled if there was a disengage controller for each subsystem. Having analysed the dynamic system in the past section and with the aim to propose a strategy of control for grasping tasks, in this section, we propose a budget for the controllers of each subsystem after being integrated into a unique vector. The error signals are created from the differences between the current state of the robot and the desired state separated by depth and attitude. The general schema for the strategy is visible in Figure 11, where all descriptions are shown.

### Acceleration Feedback Controller

According to the simplified model, the structure is similar to that of a mass-damping system, with the consideration that the terms in the damping component are nonlinear. Based on the dynamics of a mass-damping system, we can propose a controller where the main component is acceleration multiplied by the transfer function $h_{m}\left(s\right)$, the product of which, plus the output from a traditional controller such as a PD controller, can be used as a feedforward reference [33]. For this controller, we consider the control law exposed in (21), where every gain in *K* is positively defined, and the error is $\tilde{x}=x-x_{d}$:

(21) $$\tau =\underset{\mathrm{reference}\;\mathrm{feedforward}}{\underbrace{kx_{d}}}\;-\underset{\mathrm{PD}\;\mathrm{Controller}}{\underbrace{\left(K_{p}\tilde{x}+K_{v}\dot{x}\right)}}\;-\underset{\mathrm{Acceleration}\;\mathrm{feedback}}{\underbrace{h_{m}\left(s\right)\ddot{x}}}\;$$

**Figure 12:**
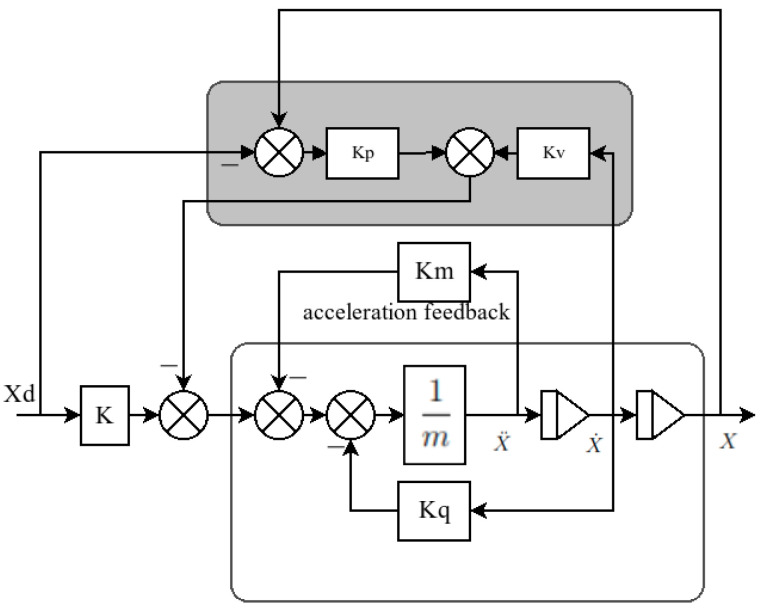
Controller schema based on the control law exposed in (21). On the bottom side, as an inner loop, is the acceleration feedback, and on the top side is a PD outloop.

For this strategy, $h_{m}\left(s\right)$ was selected as $K_{m}$ based on improving the simplicity of the control law. The controller schema can be seen in Figure 12.

The choice of values for each gain depends on the physical values, such as inertia, hydrodynamic coefficients, or mass. To summarise, the gain selection process is described in Algorithm 1, where we show the process that we followed to find the values for each controller.
**Algorithm 1** PD and acceleration feedback pole-placement algorithm**Require** **:**$\omega_{b}>0$ and ζ>01:$\omega_{n}=\frac{1}{\sqrt{1-2\zeta^{2}+\sqrt{4\zeta^{4}-4\zeta^{2}+2}}}\omega_{b}$2:$K_{m}=I_{ii}$3:$K_{p}=\left(m+K_{m}\right)\omega_{n}^{2}-K$4:$K_{v}=2\zeta \omega \left(m+K_{m}\right)-K_{qi}$

Algorithm 1 requires values for the bandwidth $\omega_{b}$ and for the relative damping coefficient ζ, which can be obtained from the characteristic behaviour of a second-order system. *K* is the feedforward gain, $I_{ii}$ is the component of the inertial tensor for each axis, *m* is the mass, and $K_{qi}$ is the quadratic coefficient for each axis.

## 5. Experimental Methodology

The experimental methodology aims to validate the use of the control strategy proposed in this paper to control an underwater robot in a grasping task. The grasping task was conducted in a test pool with the dimensions 4m×2m×2m. In this section, we will describe the experimental procedure for validation and the object to be grasped.

### 5.1. Experimental Procedure

**Figure 13:**
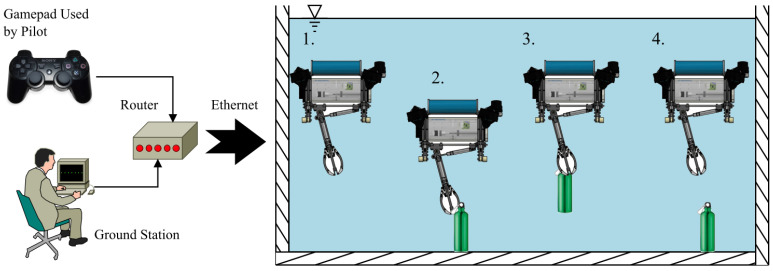
The proposed experimental procedure, where it is visible how the robot began from the ground station. The robot went through four stages: 1. the first was starting to submerge, 2. the second was taking the object, 3. the third was taking off at different depths, and 4. the last was carrying the object and releasing it in a different place.

For the experiment, we propose a sequence that involves the robot being guided by a pilot from the ground station while trying to take one object located on the pool bottom. The objects were located 1.8 m deep. The robot began the sequence from the surface or from a position higher than the object. The approach to each object was manual, and other sources of information were used, such as cameras, but every time, the control of the robot was manual. The schema of the experimental methodology can be seen in Figure 13, and was carried out for each of the objects to take.

For every case, the initial position could be different, but the experimental procedure was kept the same for each selected object. Four different stages were planned, described as follows:

1. In this first stage, the robot could be on the surface or at another depth, and will descend to approach the object.
2. For the second stage, the ability of the pilot to control the robot could improve the outcome of the grasping task, but in this case we evaluate the capability of the control to achieve the objective of taking the object.
3. When the robot has taken the object, the pilot commands taking off. At this third stage, the control of the robot is evaluated to guarantee the desired depth. Carrying the object, the robot turns around in the pool without a specific route.
4. In the final stage, the pilot makes a decision about where to release the object, and the reaction of the robot when conducting the load mass release allows the control behaviour to be analysed.

To finish the experiment, it was not necessary to complete any particular path, or to hold the object for any length of time. The comparison between different releases was carried out with parameters such as the overshoot, responses to possible disturbances, errors in the steady state, or ability to carry the object. The elements were assessed for their ability to be held by the gripper, the functionality of which has only has two options: opened or closed. The decision to open or to close the gripper is made by the pilot.

### 5.2. Objects to Take

The selected objects for these experiments were a ball, a bottle, and a plant. The robot has a gripper which is able to hold any of these objects. The operation of the gripper is controlled by the pilot, who selects when the gripper is open or not. The physical characteristics are shown in Table 1, and the described methodology applies for every object.

In the case of the ball, its diameter was 0.06 m, the outside material was plastic, and inside it there were four stainless steel balls, which weighed approximately 0.1 kg in total (each steel ball weighed 0.03 kg approx.). The ball was wrapped in a red plastic to provide contrast in underwater conditions. The used bottle was a green aluminium bottle with a diameter of 0.07 m, containing sand to increase its weight, with a plastic pipe on top to improve its manipulation. The plant was not real and was instead made of plastic, being 0.5 m in height and 0.4 m in diameter approx., with a mass of 0.31 kg. Its volume was not measured.

## 6. Results

The results of the test will be described in this section. For comparisons between the results, we used the value of the root mean square error (RMSe). Furthermore, for each situation, we describe the specific characteristic of the response, considering how the overshoot changes the reference.

For every experiment, the first approach of the depth controller, the value of its error, its behaviour, and its response to disturbance are given. The description of every test includes detail about the orientation, where the Euler angles are compared against the reference, according to the dynamic model, the angles of which allow the robot to move in the X–Y plane in an inertial-fixed frame. It is assumed that with a greater yaw angle, there will be a bigger error due to the cable torque that is not measured.

The behaviour of the robot using the control strategy during this experiment can be appreciated in the following video: https://youtu.be/EZIGtl6GJSg (accessed on 14 November 2022).

### 6.1. Results of the Ball Test

**Figure 14:**
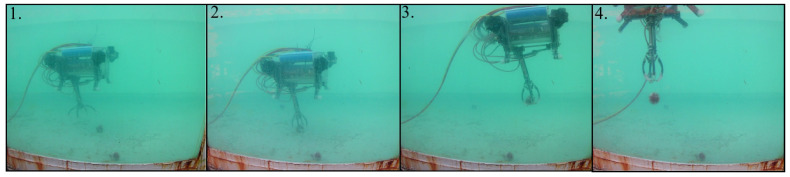
Practical results of catching the ball. The numbers are given according to the proposed stages in the methodology.

For the test that involved the catching of a ball, the elapsed time was approximately 8 min. The proposed sequence given in the methodology that was used for this experiment is visible in Figure 14.

#### 6.1.1. Depth Controller

**Figure 15:**
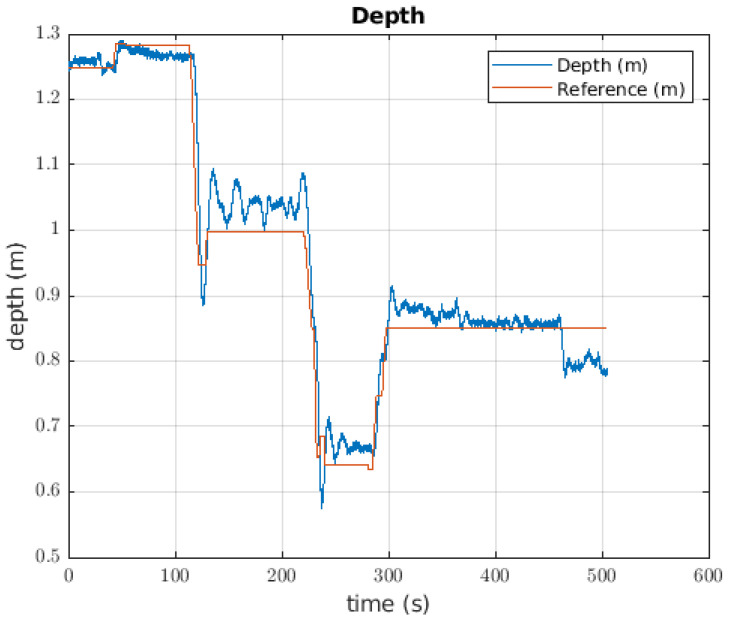
Response of the depth controller when the robot takes the ball.

The depth controller to take the ball had an error of 0.0432 m. For each change in the reference of almost 0.5 m, there was a overshoot of approximately 20%; however, when a change to the reference was made, the pilot changed the value. This effect occurred for the two first large changes in the reference that happened. Before the 120 s mark, the robot did not carry the ball, but after that time, the robot had the ball mass as its load mass. The oscillations when the reference was constant reached 0.1 m. Around 460 s, the ball was released, and the change between the reference and the current depth of the robot was obvious. This behaviour can be seen in Figure 15.

#### 6.1.2. Attitude Controller

**Figure 16:**
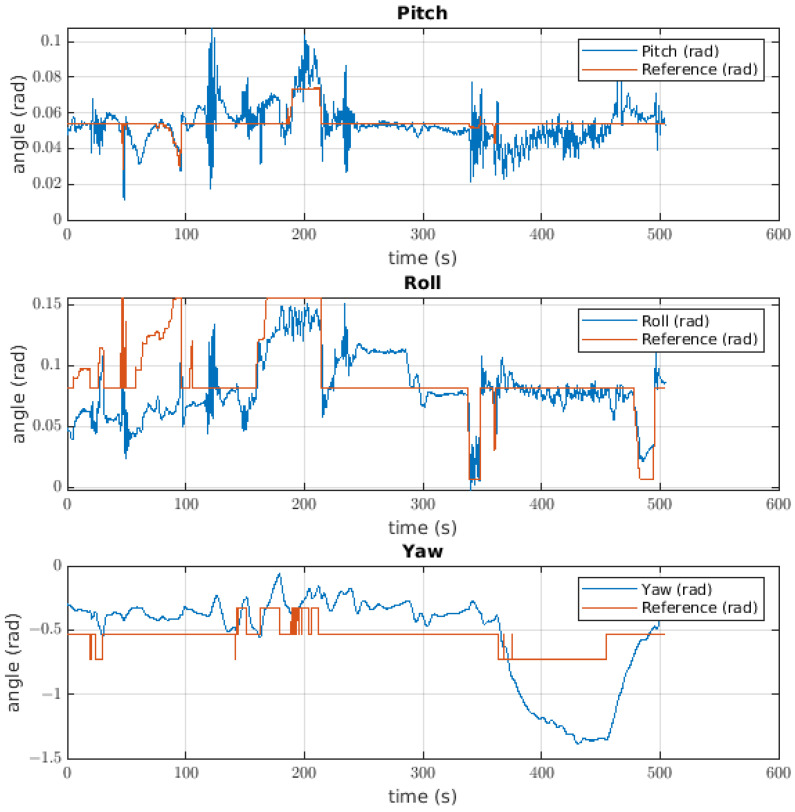
Euler angle behaviour for the attitude controller of the robot taking the ball.

The response of the attitude controller to the ball catching is shown in Figure 16, where it is possible to see the response for each angle. The first was the pitch angle with an error of 0.0133 radians. In this case, the signal noise had a magnitude of approximately 0.08 radians, but specific moments during the test showed a reference change and a change in the value of the angle; such a disturbance can be seen at approximately 460 s. The second angle was that of the roll, where the first impression is that the value of the angle has a negative offset with respect to the reference. In this case, the error was approximately 0.0407 radians, and the response of the robot was in line with the previously mentioned offset. There was a disturbance in the pitch angle close to when the ball was released. The most different signal for the Euler angles was the yaw response, where the error was 0.3474 radians and the angle changed in response to the reference changes, although these changes were not significant.

### 6.2. Results of the Bottle Test

**Figure 17:**
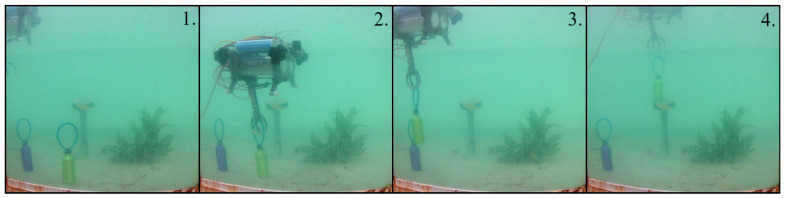
Practical results of catching the bottle. The numbers are given according to the proposed stages in the methodology.

For the case of the robot catching the bottle, the elapsed time was approximately 11 min. The proposed sequence given in the methodology that was used for this experiment is visible in Figure 17. Four tries were necessary to catch the bottle because the bottle had a rope on its top and the gripper had to close with that rope between its fingers in order for it to be caught. In the supplementary video, it is possible to see these tries. If another method of catching the bottle is used, it could be possible to use fewer tries.

#### 6.2.1. Depth Controller

**Figure 18:**
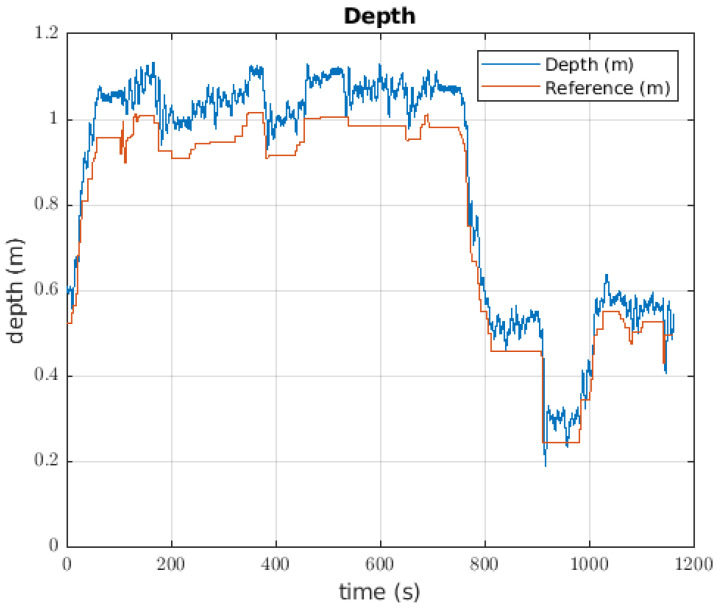
Robot behaviour when the depth controller was used to catch a bottle.

The depth controller response when the robot was used to catch a bottle can be seen in Figure 18. The error of the depth controller was 0.0822 m. In this case, the robot began from an intermediate position, but there was constant error between the signals. The robot took the bottle in approximately 700 s, from which point the pilot commanded the robot to rise until approximately 0.5 m of depth, changing the reference twice more. It is evident that the response of the robot follows the reference, with a slight error.

#### 6.2.2. Attitude Controller

**Figure 19:**
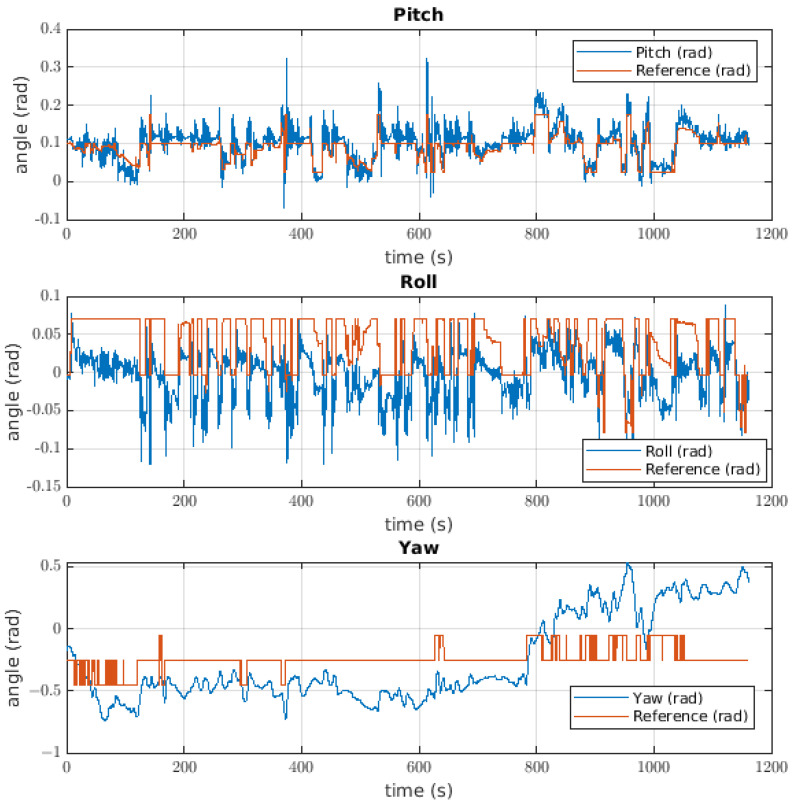
Attitude controller used by the robot to catch a bottle.

The response of the attitude controller is visible in Figure 19. For the pitch controller, the error was around 0.0527 radians, with values for the overshoot of close to 0.3 radians, but the controllers recovered their position faster. The behaviour of this controller was significant since the response of the robot was so close to the reference, although there was noise for the controller at this angle. The response of roll control has the same problem as the controller for ball catching, there being a negative offset when the error was 0.0659 radians. With this response, it is not possible to determine if the angle was controlled, but if it is possible to determine that the response follows the reference, it is important to discuss this response characteristic. The yaw response has the same behaviour as the ball yaw controller when the response of the robot does not exhibit a relationship with the reference. In this case, we must take the value of the cable torque; the response had an error of approximately 0.3323 radians.

### 6.3. Results of the Plant Test

**Figure 20:**
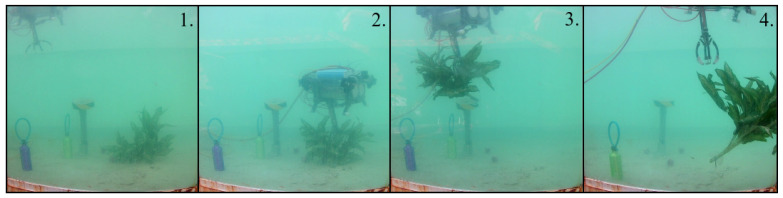
Practical results for catching the plant. The numbers are given according to the proposed stages in the methodology.

When the robot was used to catch the plant as an experiment to validate the controller, the elapsed time was more than 12 min. The proposed sequence in the methodology that was used for this experiment is visible in Figure 20. Four tries were necessary to catch the plant.

#### 6.3.1. Depth Controller

**Figure 21:**
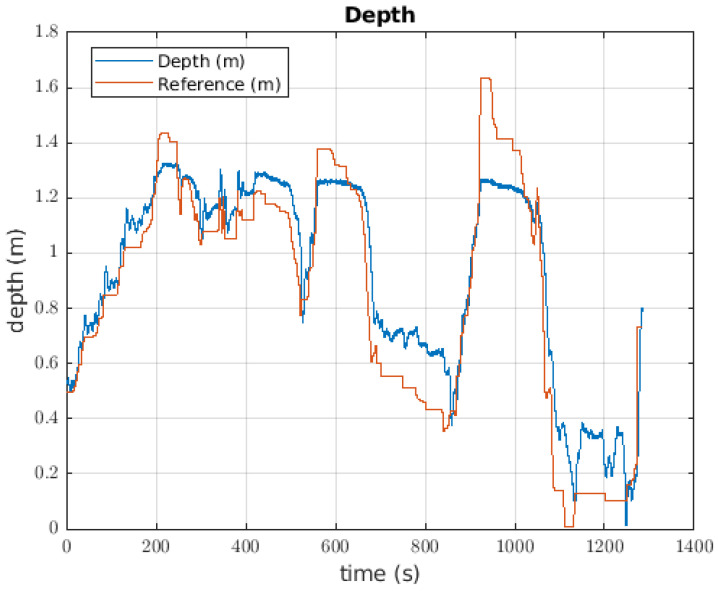
Depth controller used by the robot to catch a plant on the pool bottom.

When the robot was used to catch the plant, the response of the depth controller had an error of approximately 0.1572 m. From the 630 s mark, the response of the robot had a greater error than before; thus, this was the point at which the robot took the plant. In terms of the response of the depth controller, this was the one with the greater error value. There were two parts of the response where the error was higher, generally occurring when the robot was moving upward. The behaviour of the robot under the depth controller is visible in Figure 21.

#### 6.3.2. Attitude Controller

**Figure 22:**
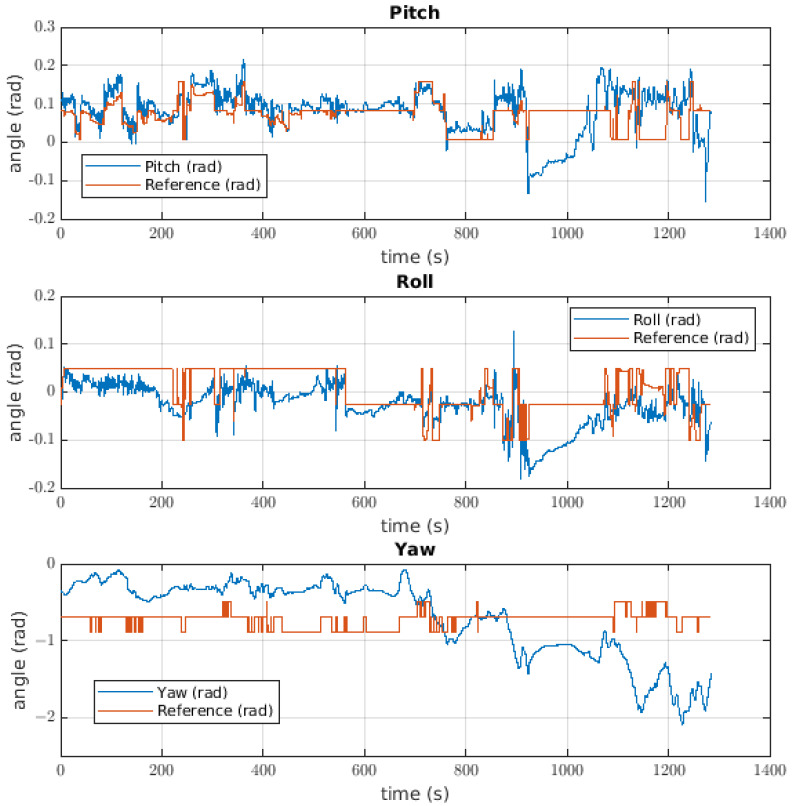
Attitude controller used when the robot was taking a plant.

The response of the attitude controller is seen in Figure 22. The first response is that of the pitch angle when the error was 0.0728 radians. For a moment, the response followed the reference, but there are parts where the response had a bigger value and the controller moved the robot to resolve the error. At the end of the experiment, the error became higher, for, in that instant, the robot continued to carry the plant. The roll response had an error of 0.061 radians, and its negative offset was visible in the controller. The values exhibit a slight tendency toward following the reference; for over 900 s of the experiment, the roll response had similar behaviour to the pitch controller. The yaw had an error of 0.5164, which was the highest error across every experiment. For this test, it is not possible to determine whether the control works at this angle to carry the plant.

## 7. Discussion

### 7.1. Implementation of the Controller

In this article, we show the results for different elements taken from the pool bottom, and provide a description of these results for each case. The metrics used to draw comparisons between them are RMS error and physical characteristics. Regarding the proposed structure of the control, its implementation was relatively easier than that of other kinds of control structure, which means that the controller could have a complex structure, but its implementation could be achieved without a problem. Measurements, such as stabilisation time and the shape to be reached, were more difficult to take, possibly due to the dynamics of the robot, which is faster than other kinds of systems. The method for calculating the gain is based on the model, where the hydrodynamics parameters were fully fundamental. If there are modelled values that vary with respect to the real values, this allows the control response to be different. It is possible that the disengagement of the model is the best way to design a controller around one point, but if the model is carried to a nonlinear zone, the robust controller will provide evidence that the system could be not controlled. The analysis of robust controllers is required to achieve a better response to disturbance for this kind of controller.

According to our experimental results, the proposed control strategy improved the position error values compared with the literature [11]. However, under simulation conditions or testing with filters such as EKF, the performance of the error signal could be worse compared with [28]. The values of error obtained with elements such as the plant were greater due to the volume and mass of the object to be caught. The absence of gravity compensation could be one of the error source to catch objects, then to include the gravitational effect into the controller could improve the performance in the task.

### 7.2. Catching the Ball

Figure 15 shows the behaviour when the ball, which is the lighter object, is picked up by the robot. Even though the robot has a suitable performance, the controller is not able to follow the given reference. A potential solution to catch these objects could be to consider the value of mass as a disturbance, or as a parameter to refresh the controller.

The pitch controller used to catch the ball gives us a response with high values of noise; these values reached up to 0.8 radians in magnitude. The value of noise could be neglected if the value of the inertial tensor for this axis is able to break the effect of inertia generated by the controller, taking into account that the controller uses the inertial value as gain for the acceleration feedback. With respect to the shape of the response, the different disturbances could come from catching the ball. For the roll response, the negative offset described in the results is possible because the centre of mass is not contemplated as an assumption, meaning that the centre of mass is moved leftward and there is a load torque. This is possible due to the disengagement of the model because the error in the middle of the test was lower than when the ball was not caught. The yaw control response has some correspondence with the reference, with a greater value of error. It is possible that the main source of this error is the cable torque, which is not measured, the value of which could be higher than that given to the system by the controller. To solve this issue, there are two solutions: the first is to use a controller with an adaptative component to make the system more robust, and the second is to identify the cable torque to be added to the model so that the controller has the ability to manage its effect.

### 7.3. Catching the Bottle

In the case of the bottle, the depth controller took longer than it did for the ball experiment. The value of noise was higher than the other tested depth controllers, and the error in the steady state was also higher, but the possible source of error was not visible. The system is controlled because the signal follows the reference; in this case, the load mass is higher than that in the ball experiment. The possible solution could be to include the value of the load mass, which is yet to be proposed for the ball experiment.

The attitude controller used to catch the bottle shows the pitch signal to have the least error of all experiments; however, the possible source of error for this case is based on the value of inertia. The roll response keeps the error shown in the ball experiment, and the same level of noise is also present. For the yaw controller, the same response happens as for the ball experiment.

### 7.4. Catching the Plant

In terms of the response of the depth controller in the case of catching the plant, the error was bigger than in the other cases, which is possibly due to the relationship between the loads. The plant has a relative density bigger than that of the bottle or the ball, and this object was more complicated to grasp for the robot. When the pilot gave the order to take it off, the motor did not have enough power to reach the reference. It is possible that if an integral component was used, the error in the steady state would be lower or even zero, but achieving this requires that the power of the thrusters is higher.

The response of the attitude controller in terms of the pitch angle showed two moments when the response was dissimilar to that of the reference. One possible reason could be the effect of the load at this angle; if the controller knew that the load had been caught, the gains could be modified and refreshed. The roll response exhibited the same problem, meaning that the controller did not contemplate the possibility of there existing an error in some assumptions.

## 8. Conclusions

It was possible to implement control based on the feedback of the acceleration, which allowed tests to be conducted with the robot in the form of a drone in an underwater environment. The development of three different tests was carried out: grabbing a ball, grabbing a bottle, and grabbing a plant.

Results were obtained for the three tests in terms of the dimensions and position of the robot. The analysis of the errors allowed us to conclude that it is valid to use this type of control to grab things in underwater environments. However, issues such as robustness and the response to disturbances are not eliminated with a controller like the one discussed in this article.

On the other hand, it can be seen that the UDrobot is a controller validation platform for underactuated robots used in underwater conditions, and the environmental conditions where the tests were carried out allow the generation of useful research products.

## Figures and Tables

**Table 1:** Physical properties of the submerged objects used for the experiments.

	Ball	Bottle	Plant
*mass* (kg)	0.121	0.770	0.31
*volume* (m^3^)	0.000112	0.000750	-
*Image*